# A complementary medicine student-led telehealth clinic: evaluating learning & teaching perceptions

**DOI:** 10.1186/s13104-024-06728-5

**Published:** 2024-03-05

**Authors:** Tracelee Shew, Catherine Smith, Greg Connolly, Michael Fleischmann, Craig S. McLachlan

**Affiliations:** 1 Health, Torrens University Australia, 17-51 Foveaux St, Surry Hills, 2010 Sydney, Australia; 2 Health, Torrens University Australia, 196 Flinders St, 3000 Melbourne, Australia; 3 School of Health & Biomedical Sciences, RMIT University, 124 LaTrobe Street, 3000 Melbourne, Australia; 4 Centre for Healthy Futures, Torrens University, 17-51 Foveaux St, Surry Hills, 2010 Sydney, Australia

**Keywords:** Telehealth, Complementary medicine, Naturopathy, Nutrition, Chinese medicine, Clinical education, Pandemic

## Abstract

**Objectives:**

This study evaluates a multi-centered complementary medicine (CM) student-led telehealth clinic during the COVID-19 pandemic. Likert and qualitative responses explore student and educator learning and teaching perceptions of the implementation of a successful telehealth clinic.

**Results:**

51 students and 17 educators completed the survey. Respondents agreed that support from educators (90%) and orientation (70%) assisted effective performance. Over 90% (93%) of all respondents supported telehealth in student-led clinics, whilst 87% encountered barriers such as technical and infrastructure issues. Respondents agreed that telehealth practice skills improved in case history taking (90%), treatment (90%) and building patient rapport (60%). Respondents (61%) disagreed that physical examination was effectively performed, and 100% of respondents agreed telehealth was a valuable learning experience. This study is the first to explore student and educator perceptions of telehealth in an Australian University multi-centered CM student-led clinic. To be successful in an educational environment, students and educators require digital literacy and adequate telehealth practice infrastructure. Whilst some in-person practice skills are transferable to telehealth, educators need to adapt curriculum to ensure counselling and physical examination skills are specifically taught for virtual consultations. Telehealth in clinical practice requires continued investigation and educational development.

**Supplementary Information:**

The online version contains supplementary material available at 10.1186/s13104-024-06728-5.

## Introduction

Complementary Medicine (CM) includes non-biomedical health disciplines such as Naturopathy, Clinical Nutrition, Western Herbal Medicine, and Chinese Medicine. CM is widespread with university trained practitioners [1–3] aligning to Australian national health priorities supporting biomedical care [4–6].

Telehealth is technology facilitated healthcare, provided at a distance, that was essential during the COVID-19 pandemic lockdowns for reducing the risk of infection in clinical settings [4, 5, 7, 8]. CM clinical educators at Torrens University Australia (TUA) implemented telehealth programs that aligned with accrediting body approvals [6, 9–11]. A unique virtual consultation approach evolved allowing students to engage with their patients from home-based and on-site telehealth clinic locations.

Pre-pandemic, TUA telehealth was not included in CM curriculum or student-led practice. Previous research indicated CM educators feared inadequate patient care with virtual consultations [12]. In private CM practice less than 10% of practitioners used telehealth [13, 14]. Despite this, CM students expressed interest in using virtual consultations in future practice [13]. During the COVID-19 pandemic, the rapid uptake of telehealth virtual consultations resulted in increased research exploring educational practices in medicine and allied health [15–18]. There is currently minimal research exploring CM education [13]. A pilot study from TUA on a naturopathic student-led home-based telehealth clinic highlighted increased peer and collaborative learning, expanded patient diversity, improved digital skills, and responsive supervisor feedback [8]. Poor technology infrastructure and difficulty with physical assessment were detractors to the telehealth learning experience early in the COVID-19 pandemic [8].

This study is an expansion of the pilot evaluation which discussed the concepts of telehealth and its emerging need with COVID-19 [8]. Our aims are to examine CM telehealth learning and teaching experience in a multi-disciplinary, multi-centered student-led clinic. The research will describe the evolution of telehealth virtual clinic programs and evaluate student and educator perception and experiences.

## Methods

### Telehealth

A hybrid clinic delivery was developed according to differing State (New South Wales, Victoria, and Queensland) public health orders, that limited patient attendance in person. The hybrid clinic consisted of students and educators who participated in telehealth clinics on campus or in home-based telehealth consultations. When on campus, students and educators observed Government COVID-19 safe health practice guidelines for hygiene and infection control procedures.

Groups of about six students from the same health discipline and one clinical academic were allocated to a 6-hour clinic session for 12 weeks. Comprehensive guidelines were developed for student and educator induction. National virtual telehealth induction training was given to all educators and students before clinic started. Long-term support from the Academic Clinic Leaders and technical specialists was available.

### Digital telehealth

Digital infrastructure met Torrens University data security and home-based confidentiality requirements. The infrastructure included a newly launched medical-grade digital patient management system (MediRecords) with built-in video-conferencing platform (Coviu). Existing platforms and Microsoft Office 365 were used for encrypted communications, and Blackboard Collaborate for multi-person observation of consultation.

### Study design

A survey for educators and students, in Additional file 1: Table S1, developed by the research team, was based on previous findings [8]. The current survey examined a range of quality markers for telehealth delivery. In February 2022, the data collection researcher sent participants a link to an electronic Qualtrics survey via university email.

The study was approved by TUA Human Research Ethics Committee (Project ID 124). All participants were informed about specifications of the study, including an ability to opt-in. The survey contained a 5-point Likert scale for quantitative questions. Associated qualitative questions provided further opportunity for participant written feedback.

### Participants

University students enrolled in a clinical practicum subject of the Bachelor of Health Science degrees for Naturopathy, Nutrition, Chinese Medicine, and Western Herbal Medicine, along with educators employed to supervise clinical students, were recruited. A total of 343 participants were invited to complete the survey, with 74 respondents, students (75%) and educators (25%), completing the survey. Respondents comprised of naturopathy (*n* = 41), nutrition (*n* = 22), Western herbal medicine (*n* = 9) and Chinese medicine (*n* = 8), with some in dual degrees or teaching over multiple programs (*n* = 6). Telehealth consultations were provided to 1,075 patients across three State-based student-led clinics during Trimester 3 (September to December) 2021. Observing preclinical students and clinical administration staff were excluded from the survey. The data collector was blind to any identifying information.

### Data analysis

Data was exported from Qualtrics, collated, and cleaned using Microsoft Excel and imported into SPSS (version 28.0) for analysis. Frequency distributions and percentages were used to describe categorical or ordinal data. The available response categories (‘strongly agree’, ‘somewhat agree’, ‘neither agree nor disagree’, ‘somewhat disagree’ and ‘strongly disagree’) were ordinal and converted into numerically weighted scales. For summary reporting purposes, the combined percentage of responses in the ‘strongly agree’, ‘somewhat agree’, and ‘somewhat disagree’ and ‘strongly disagree’ were combined into two categories respectively, to present an easily understood percentage of agreement or disagreement. Descriptive statistics were generated for each variable on the questionnaire. Qualitative data was thematically analysed by two authors and when needed disagreement was reviewed and explored by a third researcher.

## Results

### Respondent characteristics

Across the Torrens University student-led telehealth clinics, Melbourne clinics implemented home-based and on campus telehealth clinics (60%), while home-based telehealth clinics were used in Sydney (22%) and Brisbane telehealth clinics remained entirely on campus (18%). Of the survey responders, three quarters were students, 48% were in the 20–39-year age range, and overall, predominantly female (*n* = 61). Student participants (*n* = 48) reported having completed some form of clinical practice hours prior to the telehealth clinic. Although four programmes were assessed, student naturopath and nutrition students provided most of the collected responses.

### Teaching and learning perceptions

All participants agreed that telehealth was a valuable clinical learning experience, with 93% supporting the implementation of telehealth in student-led clinics. Students and educators included telehealth being convenient for the patient and an important option during lockdown. The main barrier (87%) for the use of telehealth was technical issues experienced by students, educators, and patients online. However, there was an overall significant reporting of telehealth student-led clinics enabling equitable access to health care (93%). Examples of thematic responses are included in Table 1.

**Table 1:** Thematic analysis of student and educator responses

Themes	Student responses	Educator responses
**Valuable clinical learning experience**		
Important for successful practice career	33%	41%
Developed new practice skills	29%	37%
Broader client base	23%	11%
Continuity of practice during pandemic	15%	11%
**Encountered enablers to telehealth**		
Improved diversity of clients	35%	14%
Travel, time, convenience, comfort, cost	35%	48%
COVID-19 restrictions & isolation	21%	24%
Quality of learning environment & supervision	9%	14%
**Encountered barriers to telehealth**		
Internet connectivity	43%	35%
Online clinic infrastructure difficult to manage	33%	30%
Technical issues with virtual case taking	24%	35%

**Fig. 1:**
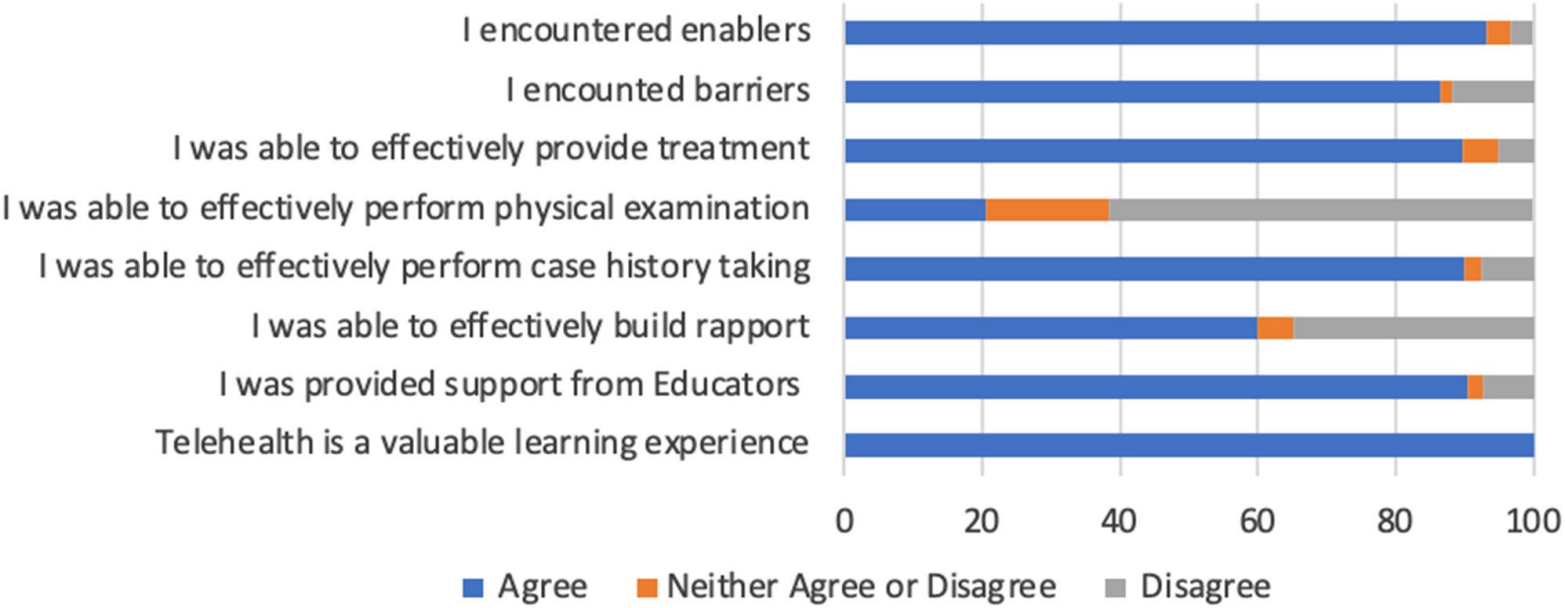
Students provided feedback on their experience in telehealth clinic

Student and educator orientation training for the induction to telehealth improved the understanding of telehealth requirements for more than two thirds (70%) of participants. However, some students (9%), reported that orientation did not prepare them adequately. While 90% of students answered positively on educator support during their telehealth experience (Fig. 1), there was much lower agreement for perceived long term telehealth support. Most respondents (80%) agreed telehealth increased opportunities to collaborate with peers and educators.

Nearly three quarters (72%) positively reported the patient came into the telehealth consultation understanding what to expect. When assessing the consultation process, 90% agreed that case history taking was effectively performed in a telehealth setting, with students suggesting limited differences in telehealth to in-person. Digital student case notes during the consultation were reported as manageable (82%).

An aspect of telehealth found to be a disadvantage for the students was the ability to perform physical examinations on the patient (61%). In-person physical exam training, provided to students pre-COVID, was reported by half the participants (53%) as not being transferrable to telehealth. More than half (60%) agreed rapport could be effectively built in an online setting. There was a positive response (90%) for the use of telehealth to provide treatments (Fig. 1). Participants reported using external dispensaries for telehealth patient treatments useful though sometimes slower for products to be delivered.

## Discussion

This study is the first to explore student and educator perceptions of telehealth in a multi-center CM student-led clinic. The main outcomes relate to the themes of value, benefits, challenges, and practice competencies.

### Value, benefits and challenges

Results indicated that value, benefits, and challenges of telehealth remained consistent with the previous pilot study [8]. Respondents perceived continuity of practice, future career success and diversity of patients as important. Similar evidence was found in medical and allied health studies on increased access to vulnerable, rural, and remote populations [19, 20]. Our study found respondents perceived patient isolation during the pandemic as an enabler of telehealth. This aligns with current evidence that lower social connectedness experienced during long lockdowns correlated with higher psychological distress and lower life satisfaction [21]. These factors may have influenced demand for CM services during the pandemic and further exploration of telehealth patient demographics is recommended post-pandemic.

Respondents in both home-based and on-site locations reported technology barriers. Unstable internet and insufficient broadband were perceived to interfere with case taking, whilst management of complex digital infrastructure was challenging. Compared to the pilot study, respondents expressed increased acceptance of technical difficulties and a problem-solving approach to resolve any deficits. This has been shown in studies with an acceptance of new technology improved through user involvement in systems development [22]. Our study participants revealed less resistance to telehealth issues due to being immersed in telehealth clinic orientation. However, students expressed a lack of long-term support and a need to rely on educator support to continue to navigate the learning environment. An interesting finding was that the success of the virtual learning environment was educator dependent, where students appreciated more opportunity for real-time feedback.

### Practice competencies

There are multiple factors that influence client rapport in telehealth. Virtual consulting requires a high level of student digital literacy [23, 24]. Our respondents reported competence for tasks requiring narrative skills for information gathering, treatment instructions, and patient education. There was a mixed response regarding the ability to establish rapport with the patient. Previous research suggests creating an environment of trust, where the patient feels cared for, valued, known, and enhances the therapeutic relationship [25, 26]. Studies have identified difficulties with looking at the camera whilst also managing digital case taking and technology infrastructure may impact person-centered practice [27, 28]. Respondents perceived video conference as helpful in building rapport as it allowed viewing of the patient and their living circumstances.

Conversely, in-person consulting during the pandemic required masks for infection control and was regarded by respondents as a detractor to development of interpersonal connections.

Like our previous pilot telehealth study [8], challenges were reported in the ability to perform physical examination in a virtual environment. Several respondents expressed frustration with not being able to perform in-person physical examinations. Thus, risk management during our telehealth program required the need for medical referrals for in-person health assessment. University curriculum updates incorporating virtual assessment techniques are necessary for safe practice [15, 27, 28]. Several studies recommend comprehensive case history taking [15] as well as patient education on self-monitoring techniques using home and wearable devices. These include apps to track activity, sleep, and food intake [29, 30] and self-assessment for vital signs and other body changes [31, 32].

## Limitations

This was a multi-programmed, multi-centred, single university study held during the COVID-19 pandemic. Outcomes may be unique and not generalisable to post-pandemic telehealth clinics. This was a cross sectional study therefore causation cannot be inferred. Some questions required previous recall, and this captured some longitudinal changes in behaviours and self-cognitions related to telehealth. Infrastructure may vary in other settings, thus there is caution in extrapolating outcomes. Telehealth may require an expanded interprofessional referral network such as performing full physical examinations, and referrals to physicians may add costs to the patient.

This study explored student and educator perceptions of telehealth in an Australian University multi-centered CM student-led clinic. A key enabler of the telehealth program at Torrens University is digital literacy. Counselling and physical examination skills are specifically taught for virtual consultations. Telehealth is highly valuable for vulnerable populations, pandemic conditions, patients in remote areas and consultations for convenience. Telehealth in clinical practice requires continued investigation and educational development. Importantly, risk management was reinforced in a telehealth program. This study provides a suitable framework for further studies to evaluate post-pandemic CM telehealth practice in a university setting.

### Electronic supplementary material

Below is the link to the electronic supplementary material.

Supplementary Material 1

## Data Availability

The quantitative and qualitative survey responses are the corresponding author on reasonable request.

## Abbreviations

CM: Complementary Medicine
COVID-19: Coronavirus disease 2019
