# Supplementary material for: A complementary medicine student-led telehealth clinic: evaluating learning & teaching perceptions
Source: BMC Res Notes. 2024 Mar 5;17:65. doi: 10.1186/s13104-024-06728-5 (PMC10913539; doi:10.1186/s13104-024-06728-5)
Supplement: Supplementary file 1 — Supplementary Material 1 [file 13104_2024_6728_MOESM1_ESM.docx]

**Additional file 1 of the Telehealth Survey**

**Table S1: Telehealth Student and Educator Survey**

1. Please select your gender
2. Please select your current role at Torrens University
3. Please write your age below (e.g., 34)
4. Please select your campus location
5. Please select the course specialisation/s you are completing as a student or currently teach into: Naturopathy
6. Please select the course specialisation/s you are completing as a student or currently teach into: Western Herbal Medicine
7. Please select the course specialisation/s you are completing as a student or currently teach into: Clinical Nutrition
8. Please select the course specialisation/s you are completing as a student or currently teach into: Chinese Medicine
9. Please select the course specialisation/s you are completing as a student or currently teach into: Clinical Myotherapy
10. Telehealth is a valuable clinical learning experience
11. I encountered barriers (e.g. internet connectivity issues; difficulty when performing required tasks of clinical consult) related to the use of telehealth
12. Please explain why you disagree you encountered barriers to the use of telehealth
13. I encountered enablers (e.g. patients were able to continue care without needing to attend clinic in person) related to the use of telehealth
14. The students find taking case notes for patients is manageable when using telehealth
15. An orientation to telehealth from Torrens University improved my understanding of the requirements for Telehealth
16. I was provided support from my educators during my Telehealth experience
17. The client came with a general understanding of what to expect in a Telehealth consultation
18. I was able to effectively build rapport with a client using Telehealth
19. I was able to effectively perform case history taking of the client during Telehealth
20. Taking case notes is manageable when using telehealth
21. I was able to effectively perform client physical examinations using Telehealth
22. I was able to effectively provide treatment on the client during telehealth
23. In-person training on physical examination of the client was transferable to a telehealth setting
24. Telehealth increased opportunities to work on client cases between student peers and educators
25. I support the use of telehealth in student clinic

Questions 11-24 contained drop down question “Please explain” for strongly agree, somewhat agree, strongly disagree, somewhat disagree agree
